# A Novel Approach to Training Monotony and Acute-Chronic Workload Index: A Comparative Study in Soccer

**DOI:** 10.3389/fspor.2021.661200

**Published:** 2021-05-31

**Authors:** José Afonso, Fábio Yuzo Nakamura, Rui Canário-Lemos, Rafael Peixoto, Cátia Fernandes, Tomás Mota, Miguel Ferreira, Rafaela Silva, Armando Teixeira, Filipe Manuel Clemente

**Affiliations:** ^1^Centre for Research, Education, Innovation and Intervention in Sport, Faculty of Sport of the University of Porto, Porto, Portugal; ^2^Research Center in Sports Sciences, Health Sciences, and Human Development, University Institute of Maia, Maia, Portugal; ^3^Associate Graduate Program in Physical Education Universidade de Pernambuco/Universidade Federal da Paraíba, João Pessoa, Brazil; ^4^Department of Sports Sciences, Exercise, and Health, University of Trás-os-Montes e Alto Douro, Vila Real, Portugal; ^5^Research Group in Strength Training and Fitness Activities, Vila Real, Portugal; ^6^Certified Strength and Conditioning Specialist, Independent Researcher, Lisbon, Portugal; ^7^University Institute of Maia, Maia, Portugal; ^8^Faculty of Engineering of the University of Porto, Porto, Portugal; ^9^Institute of Biomedical Sciences Abel Salazar, Porto, Portugal; ^10^Escola Superior de Desporto e Lazer, Instituto Politécnico de Viana do Castelo, Rua Escola Industrial Comercial de Nun'Álvares, Viana do Castelo, Portugal; ^11^Instituto de Telecomunicações, Delegação da Covilhã, Covilhã, Portugal

**Keywords:** football, performance, load direction, load management, multidimensional models, training monotony, acute:chronic

## Abstract

Load is a multifactorial construct, but usually reduced to parameters of volume and intensity. In the last decades, other constructs have been proposed for assessing load, but also relying on relationships between volume and intensity. For example, Foster's Training Monotony has been used in athletes' load management simply by computing mean weekly load divided by its standard deviation, often multiplied by session rate of perceived exertion. Meanwhile, the Acute to Chronic Workload Ratio (ACWR) has been debated by the sport scientists as a useful monitoring metric and related to so-called injury prevention. None of these models includes parameters that are representative of training specificity, namely load orientation. The aim of this study is to present broader conceptual approaches translated by new indices for assessing Intraweek Training Monotony (ITM) and Acute to Chronic Workload Index (ACWI) while incorporating load orientation, session duration and weekly density (frequency normalized) in addition to parameters related to proxies of external and/or internal load. Our ITM and Foster's Training Monotony were similar in terms of average values, but very different for individualized analysis, illustrating how average values may be deceiving. While Foster's model provided clusters of values, ITM provided more scattered, individualized data. ACWI and ACWR provided very distinct qualitative information, and the two models were uncorrelated. Therefore, the models incorporating training load orientation presented in this study provide distinct and not redundant information when compared to previous models. More importantly, ITM and ACWI are metrics that are compatible to each other and might fit to coaches' monitoring targets in the short and medium terms, respectively. Because our models include several parameters, including load orientation, we contend that might provide a more complete monitoring tool. However, we suggest they are used for intraindividual comparisons and not so strongly for interindividual comparisons.

## Introduction

Training is a multifactorial process (Bompa and Buzzichelli, 2018) where a delicate balance between load and recovery should be achieved (Kellmann et al., 2018). Such balance will hopefully allow improvements in performance while diminishing overuse injuries and drop-out rates (Schwellnus et al., 2016; Soligard et al., 2016; Aicale et al., 2018), although these issues remain complex and controversial (Kalkhoven et al., 2021). Planning has a role to play in this context (Bompa and Buzzichelli, 2018), but monitoring the actual training load and the athletes' responses is paramount (Griffin et al., 2020; McGuigan et al., 2020). While coaches' memory may be biased and partial (Brawley, 1984; Laird and Waters, 2008), an accurate recording of what was actually performed provides a more objective basis for understanding the dynamics of the process and support future decision-making. But defining what training load is and actually measuring it is a very complex subject matter (Mujika, 2017; Afonso et al., 2018) and should not be reduced to a single magical number (Impellizzeri et al., 2020a). Indeed, several parameters can be used to define training load: volume, intensity, frequency, density, monotony, orientation, complexity, among others (ACSM, 2018; Bompa and Buzzichelli, 2018; Delecroix et al., 2019).

Sports Sciences have, however, been overly focused in training variables such as volume and intensity (Bradbury et al., 2020), and sometimes frequency (Schoenfeld et al., 2019), while largely neglecting other dimensions of load (Piggott et al., 2019). Volume and intensity are important load parameters (Mangine et al., 2015), but they provide an incomplete picture. For example, training frequency seems to be an important load parameter, even when the training programs have equal volumes (Ochi et al., 2018). Research on exercise prescription has even explored the minimal effective doses for preserving endurance and strength over time (Spiering et al., 2021), but load was defined by these three parameters: intensity, volume and frequency. Although frequency has been equated with weekly density (Bompa and Buzzichelli, 2018), training density could reflect changes in rest periods, for the same workload (La Scala Teixeira et al., 2019), i.e., density can represent a measure of how compact the workload was, establishing a relationship between the number of training sessions relative to a 7-day period. In a sense, weekly density provides an assessment of frequency normalized to the week. Load complexity has also been proposed as an important composite parameter, loosely defined as reflecting the degree of sophistication or difficulty of a skill (Bompa and Buzzichelli, 2018), or as reflecting the difficulty, variability and uncertainty involved in the actions to be performed (La Scala Teixeira et al., 2019). Currently, there is no satisfactory operational definition of load complexity.

In the vein of composite load parameters, the concept of Training Monotony has slowly but steadily making its appearance in research (Delecroix et al., 2019; Clemente et al., 2020). Training Monotony (e.g., weekly) is mainly calculated in one of two manners: dividing the mean daily duration of the training sessions by the standard deviation, with or without having previously multiplied the session duration by the session-rating of perceived exertion (Foster, 1998). Therefore, the concept of Training Monotony is not reflecting the diversity of training contents. Furthermore, if weekly training sessions have similar duration and perceived exertion levels, that week will be considered monotonic, even if the specific contents and training stimuli diverged widely. In fact, even when multiplied by perceived exertion, this index is dominated by session duration (Weaving et al., 2020). Expanding the premise of Training Monotony to a larger number of weeks or even months, it is natural to arrive to models of Acute to Chronic Workload Ratios (ACWR), created with the purported goal of gaining a better insight and control over injury risk (Gabbett et al., 2016). The ACWR is calculated dividing the acute load (current week) by the so-called chronic load (usually the rolling 4-week average, or exponentially). Most studies calculating the ACWR use the rating of perceived exertion and time of session/competition in order to register the training load values, but others use total distance and distance in high-speed running (Gabbett et al., 2016; Clemente et al., 2019). Based on this data, there have been suggestions that injury likelihood increases when this ratio is above 1.5 arbitrary units (A.U.) and/or when it is low, within 0.8 to 1.3 A.U. (Soligard et al., 2016; Malone et al., 2017). However, association should not be confused with causation (Stovitz et al., 2019). Recent research has questioned the validity of using the ACWR to predict injury risk (Fanchini et al., 2018; Enright et al., 2020; Impellizzeri et al., 2020a; Sedeaud et al., 2020; West et al., 2020) and called for a re-framing of the conceptual model behind the ACWR (Impellizzeri et al., 2020b; Kalkhoven et al., 2021).

Monotony, whether applied to a single training week or to several weeks, can be understood in a broader manner, incorporating additional load dimensions, such as load orientation. Attempts have been made to categorize load orientation according to four major training factors: tactical, technical, physical and psychological (Bompa and Buzzichelli, 2018). Each major factor can, in turn, be further divided into different components. For example, exercises with a focus on the so-called physical factor can emphasize different aspects of it, such as strength, power, speed, agility, flexibility, endurance, or coordination, among others (ACSM, 2018; Bompa and Buzzichelli, 2018). Technical drills can be more analytical or more contextualized (Schmidt and Wrisberg, 2008), while tactical practices can range from tactically-driven drills to small-sided games, to full simulated matches (Sarmento et al., 2018). These classifications may be helpful for load management (Loader et al., 2012), creating an expanded framework that is not limited to volume, intensity and frequency. Indeed, we contend that load orientation plays a core role in the adaptations that are induced by training. For example, different training modalities (e.g., endurance training vs. resistance training) tend to produce differential adaptations (Werner et al., 2019; Morville et al., 2020). Moreover, different methods within the same training modality (e.g., different formats of small-sided games) may induce different adaptations (Clemente et al., 2021).

The aim of this work is therefore to propose a broader conceptual approach and new indices for assessing training monotony and acute to chronic workload. Specifically, there is an explicit attempt to integrate load orientation and weekly density (frequency normalized) into a novel load management strategy, while keeping a balance between depth and easiness of implementation without huge demands of time or technology in the field. Proxies for volume and external/internal load are also contemplated. Two models will be presented: one for assessing Intraweek Training Monotony (ITM), another to assess Acute to Chronic Workload Indices (ACWI). The goal behind both models is to deliver a new tool for monitoring training loads in a more complete, multidimensional manner, and to assist coaches in adjusting the planning. We invite researchers to conduct independent validation research concerning our proposals. We do not aim to provide the ultimate metric or the Holy Grail of load monitoring, nor will we attempt to state that our indices are in any way promoters of a reduction in injury risk. That is for the future to decide.

## Methods

### Participants

Twenty-seven professional soccer players (25.1 ± 2.9 years, 181.9 ± 6.3 cm, 73.1 ± 6.3 kg) were daily monitored over 25-week period using a microelectromechanical system [MEMS]. The inclusion criterion was that any given player could have skipped a maximum of one training session during that week and have participated in the full length of the remaining sessions. Participants were informed about the study design and methodology. The procedures were part of their daily sport activities. All of them signed a free consent about their inclusion in the study. The study followed the ethical standards of Declaration of Helsinki for the study in humans.

### Experimental Approach to the Problem

This study followed an observational analytic design. External load measures were daily collected from training sessions of professional soccer periods. The period of observation occurred between September 2018 and January 2019 (early- and mid-season). Data was originally collected for other goals and was repurposed for illustrating the new conceptual models. Data from the cohort of athletes will be used to test the new models and contrast them with the previous Training Monotony equation and ACWR.

### Data Collection and Instrument

A MEMS (JOHAN Sports, Noordwijk, The Netherlands) consisting in 10-Hz Global Positioning System (GPS) including EGNOS correction and an accelerometer, gyroscope, and magnetometer (100 Hz, 3 axes, ±16 g) were used. A previous study reported validity and reliability results of this device for monitoring external load (Nikolaidis et al., 2018). The MEMS unit was always used by the same player to reduce inter-unit variability error. The unit was placed in a custom-design bag within a vest. The unit was fixed between the scapulae of the players. The data was recorded throughout each training session, namely including the moments of warm-up, breaks and cool-down. The same observer recorded all the periods of exercises and, after that, has split the data based on those periods. The variables that was collected for each session and that was used as a proxy of internal load was total distance (TD: consisting in total number of meters covered by a player during the session/exercise). Here, as our goal was merely to present a proof of concept with a simple interpretation, and so only total distance was used, as it is an easy metric to collect and interpret.

### Previous Monotony and Acute:Chronic Models

The commonly used formula for calculating Training Monotony was originally proposed by Foster (1998). In the original proposal, session duration was multiplied by the session-RPE (sRPE), and this product was termed the “session load.” If multiple daily sessions were performed, a simple sum was performed, and a single daily value was obtained. Each week, the mean and standard deviation (SD) for this session load were calculated, and the division of the mean by the SD (i.e., the inverse of a typical coefficient of variation) provided the value for “monotony.” Our data does not contain sRPE, but as was established in the introduction, even when multiplied by sRPE, Foster's index is still dominated by session duration.

In the ACWR, the acute workload is simply a sum of each day's workload during a seven-day period. The chronic workload considers the average load of the last 4 weeks of training, and the acute week can be included in the chronic load (i.e., coupled) or excluded from the chronic load (i.e., uncoupled) (Windt and Gabbett, 2019). The coupled and uncoupled models provide very similar results (Coyne et al., 2019; Gabbett et al., 2019). For our purposes, we chose to apply the uncoupled model. For the ratio itself, the acute workload is divided by the chronic workload. If the total acute workload is divided by the average chronic workload, the model is termed rolling average (Williams et al., 2017). In this model, the weight of each workload entering the equation is equal. In contrast, the exponentially weighted moving average (EWMA) may provide a more balanced approach, as it assigns a decreasing weight to the older workload values (Williams et al., 2017). This is an uncoupled model where the acute week is divided by the chronic weeks, with a proportionality factor to provide great weight for the more recent weeks and a smaller weight for the remaining weeks. Therefore, we will adopt the EWMA model for purposes of comparison with our own models.

### Our Proposed Conceptual Models

Two conceptual models were developed: an Intraweek Training Monotony Index (ITM) and an Acute:Chronic Workload Index (ACWI). The main goal was to provide a tool to assess load dynamics that incorporates load orientation and weekly density (frequency normalized) in addition to proxies of volume and external/internal load. Table 1 presents the proposed hierarchical model for categorizing load orientation. Each training session may have one or more load orientations, depending on how the coaches organize the session. For example, the coach may start with mobility work, then proceed to speed work, followed by technically driven skills and small-sided games, and therefore this particular training session would have four different load orientations.

**Table 1:** Model for categorizing load orientation according to main focus^a^, ^b^.

**Broad domain**	**Specific domain**	**Examples**
Tactical-technical	Simulated full competition (free or conditioned)	Simulated 11 vs. 11 in soccer.
	Small-sided games (further conditioned or not)	Small-sided games in team sports.
	Tactically driven drills	Drills focused on team dynamics.
	Technically driven drills	Drills focused on improving technique, but with some degree of contextual interference.
	Analytical technical drills	Isolated practice of a given technical element or combination of elements under highly standardized conditions.
Physical	Strength	Resistance training.
	Power	Plyometrics.
	Speed	Sprint training.
	Agility	Change of direction drills.
	Endurance	Repeated sprint ability.
	Mobility	Dynamic stretching.
	Equilibrium	Dynamic balance.
	Combined/complex training	Combination of two or more of the previous categories.
Activities without physical engagement		Lectures, video analysis, imagery.
Others (items falling outside the major		—
categories)		

a *The mathematical models assess the similarities and differences between load orientations*.

b *Each training session may have multiple load orientations*.

**Figure 1:**
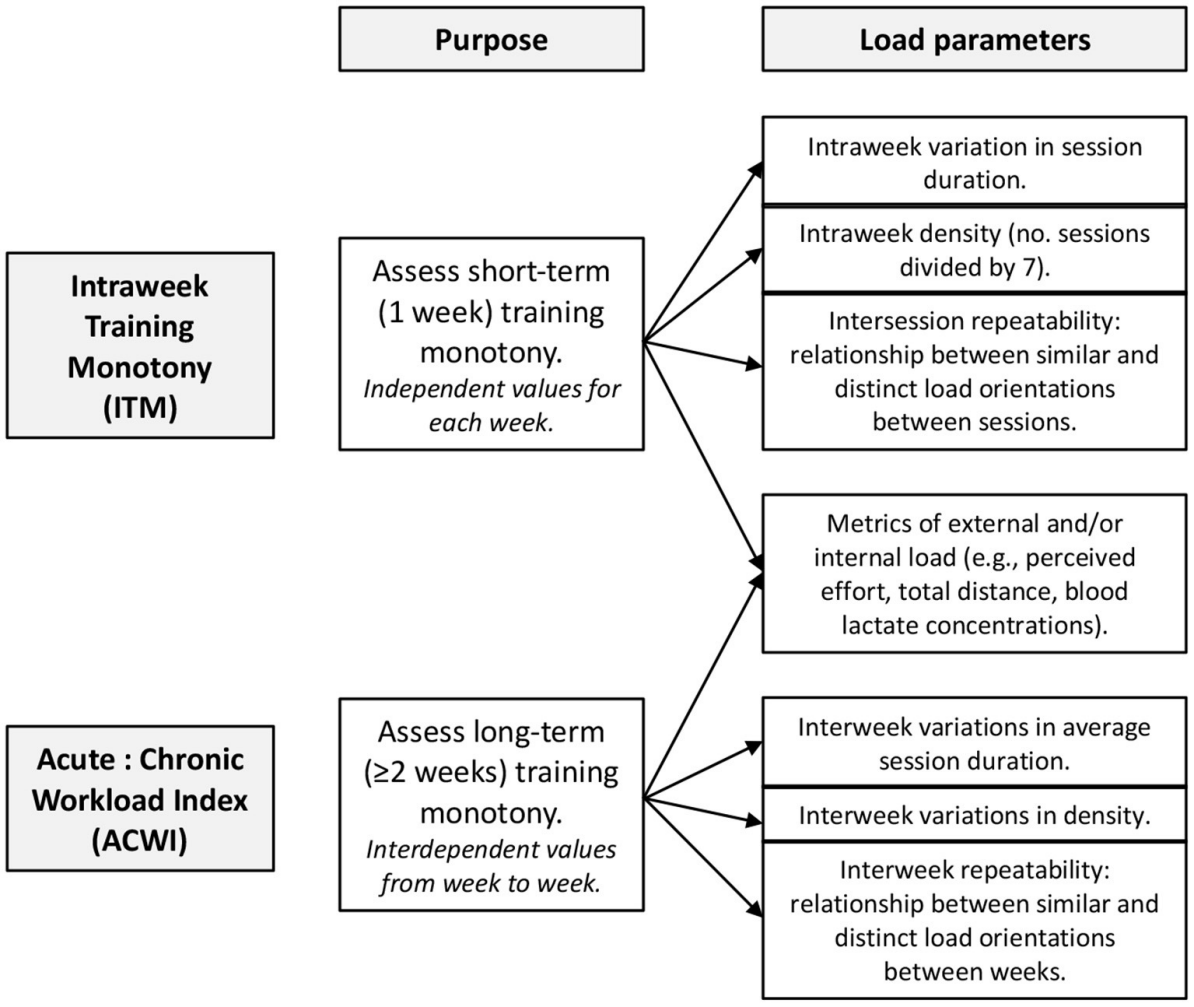
Conceptual models for Intraweek Monotony Index (ITM) and Acute:Chronic Workload Index.

The conceptual features of components used for developing the mathematical framework for ITM and ACWI are presented in Figure 1.

While ITM reflects independent values for each training week, ACWI provides interdependent values, as the current week and past weeks will have an impact on the index. Therefore, the index is dynamic and will evolve as more information is brought. However, weeks closer to the acute week will have a greater impact than weeks farther from the acute week. Both ITM and ACWI consider four parameters: (i) session duration as a proxy for session volume; (ii) weekly density (i.e., frequency normalized); (iii) metrics of external and/or internal load; and (iv) intersession or interweek repeatability, which is deeply related to load orientation. Our original idea was to attribute differential weights to the different components of the models. However, we could not find solid support on the literature and so in the current version all the factors have the same weight in the equations. Beyond the similarities, there are specificities to each model:

- *Session duration*: ITM calculates changes in session duration across one training week but does not consider days without training sessions. Therefore, variation is derived from session duration only, and not from having rest days. In ACWI, the differences in mean session duration from week to week are calculated.
- *Weekly density*: in ITM, weekly density is simply the number of training sessions divided by seven. In ACWI, the differences in density from week to week are calculated.
- *Metrics of external and/or internal loads*: (i) Although the models only accept one value for the metrics parameter, this value can originate from a single metric, or a from a combination of metrics, and is considered to reflect the load of the training session as a whole. Each coach should decide what the relevant and/or readily accessible metrics are, and how to combine them. The model will treat the value introduced by the coach but will not limit the origin of that value. This provides plasticity and means that the model can be applied to different sports and realities. (ii) ITM analyses inter-session variation of these metrics, while ACWI analyses inter-week variations. In our model, only total distance was used, to provide a simple example for the coaches.
- *Repeatability of load orientation:* Establishes a relationship between the number of similar load orientations and the total number of orientations addressed in any two training sessions or training weeks. In ITM, this reflects an average of inter-session differences. In ACWI, it reflects the average of between-week differences. On a technical note, the mathematical model actually reflects the average similarities and not the differences.

**Figure 2:**
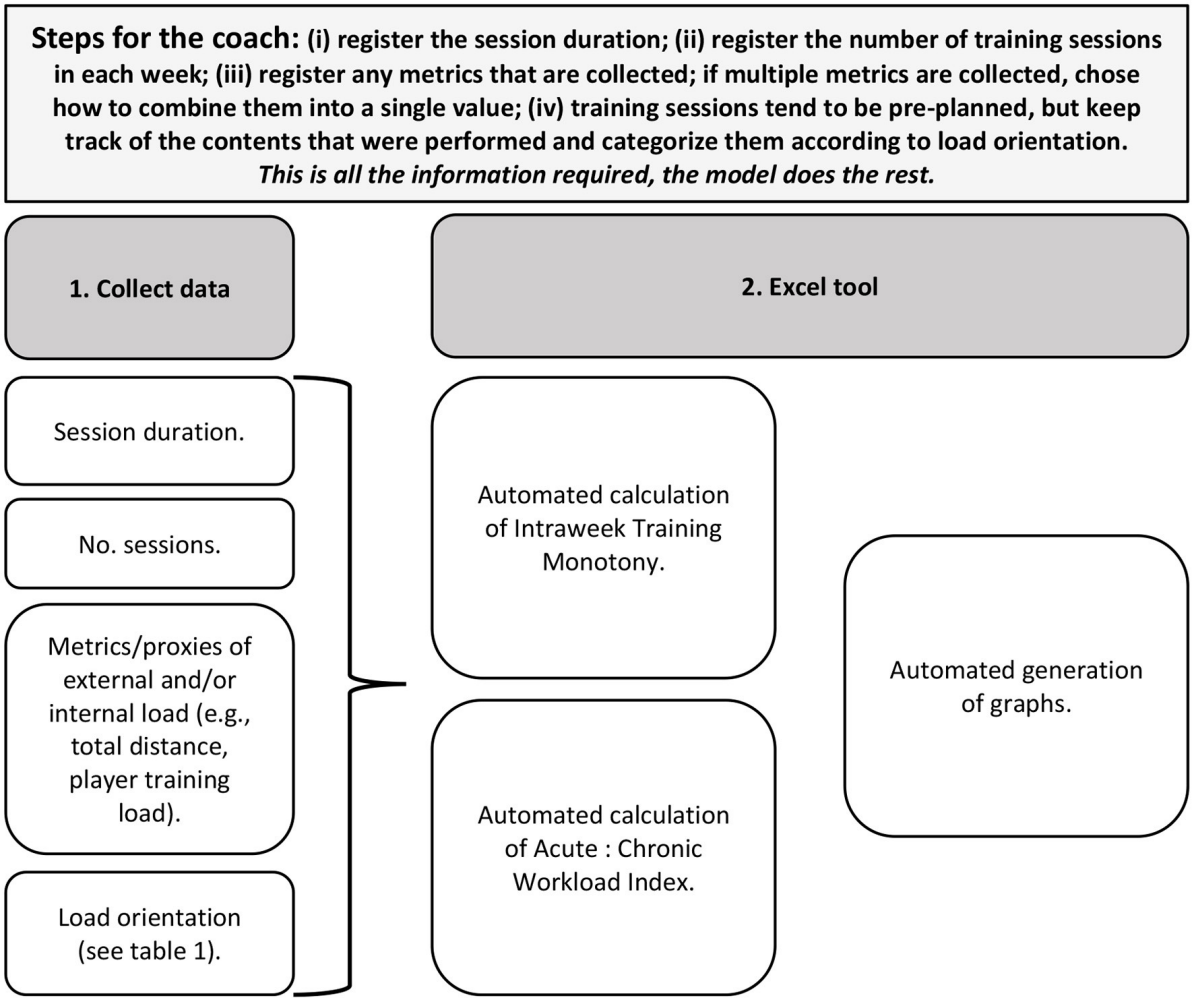
Steps for the coaches to use the models.

Figure 2 shows the steps a coach has to undertake for using the models.

### Mathematical Models

The mathematical models for ITM and ACWI are provided as Supplementary Material. All models and calculations were performed using Microsoft Excel. Due to the complexity of the models, a free Excel tool for calculating both the ITM and ACWI will be made available upon publication.

## Results

Since the goal of this work is to present new conceptual models and underline their major qualitative differences in relation to previous models, we will not present detailed data for each player. Instead, we present a simplified approach that focus on the prominent aspects. However, the original data is available as Supplementary Material. All metrics for all models are presented in A.U.

### Training Monotony

**Figure 3:**
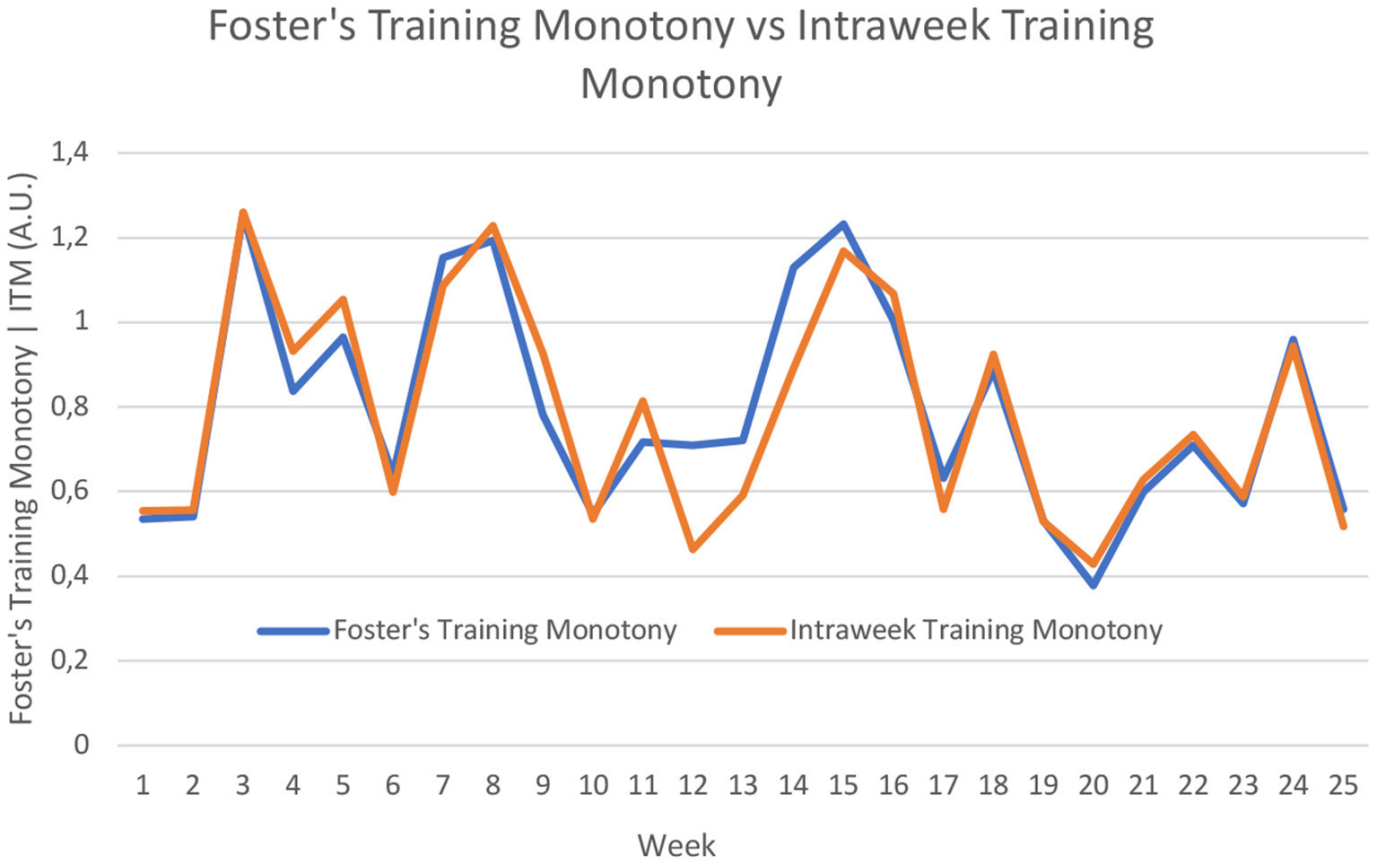
Comparison of average data between Foster's Monotony Index and Intraweek Monotony Index. A.U., Arbitrary units.

**Figure 4:**
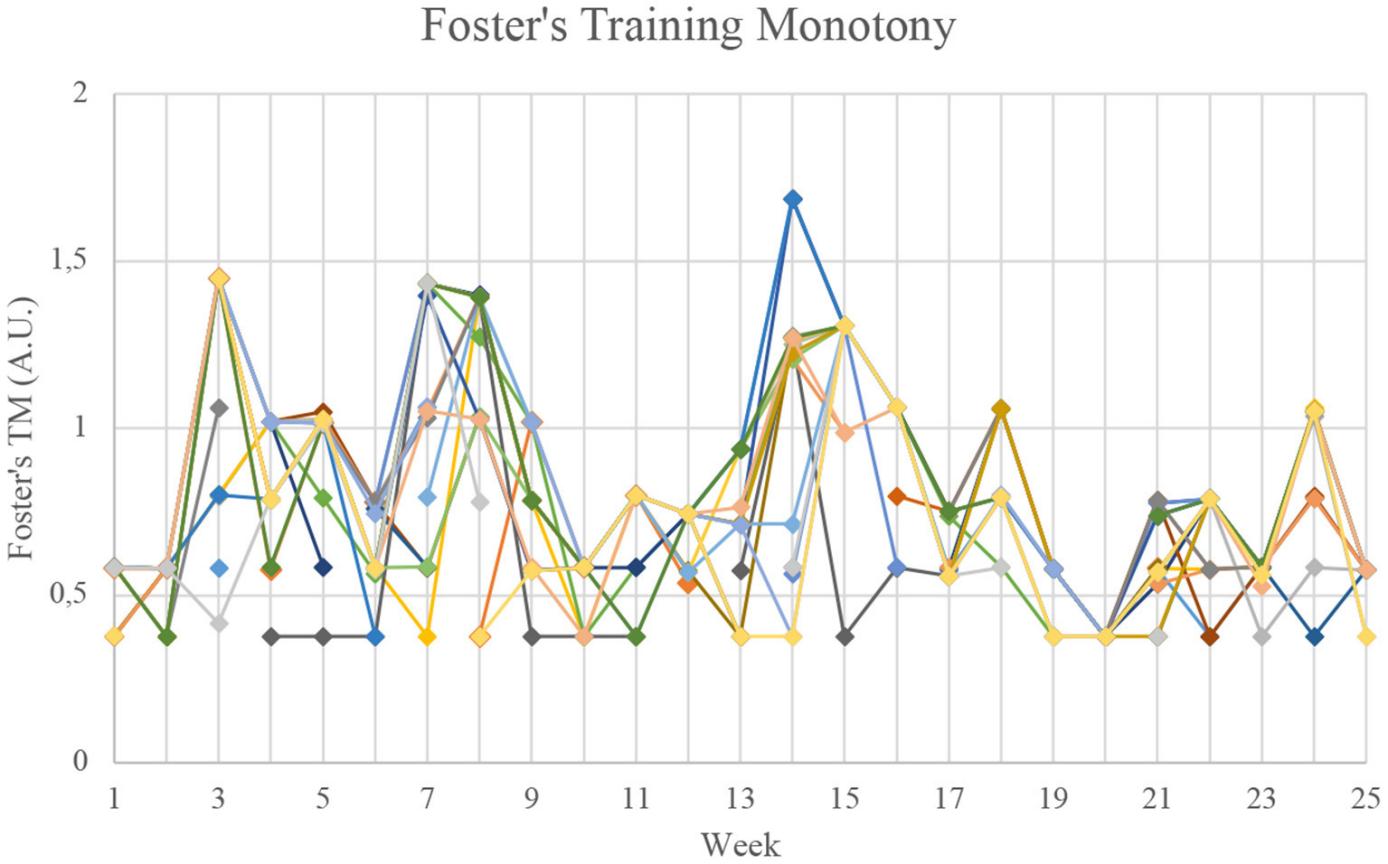
Individual values for Foster's Training Monotony. A.U., Arbitrary units.

**Figure 5:**
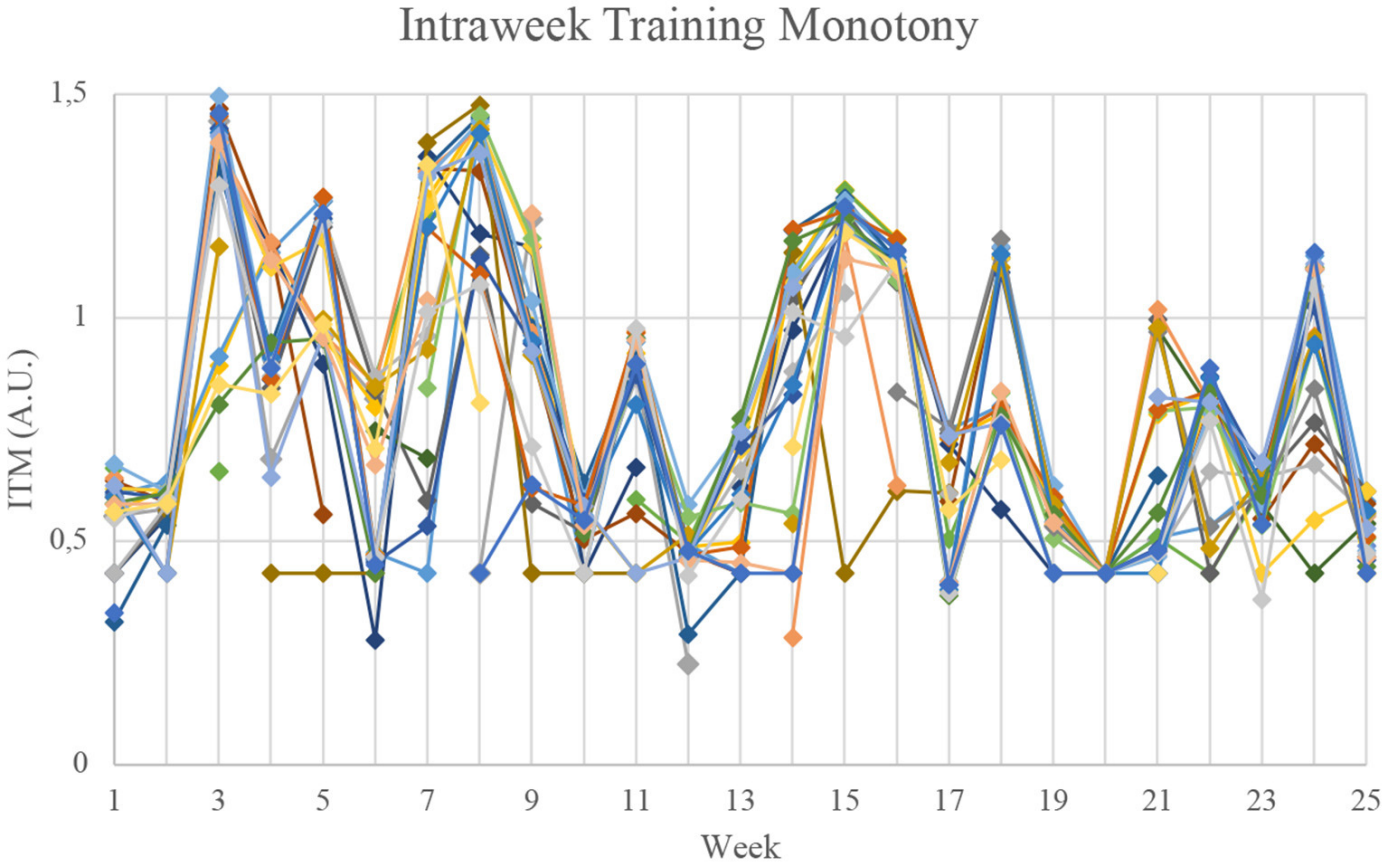
Individual values for Intraweek Training Monotony. A.U., Arbitrary units.

Figure 3 presents an overall comparison of the 27 athletes between Foster's Training Monotony and ITM, while Figures 4, 5 present the individual data for both models. The overall view of the models suggest they provide similar information (*r*^2^ = 0.85), with a few notable exceptions in weeks 12 and 14. Analyzing the raw data, it can be seen that from week 12 to week 14, there was a 32.7% average increase in total distance. There were also notable changes in load orientation. For example, in week 12, simulated full competition comprised 7.98% of the loads, while in week 14 in represented 20.06%. Conversely, analytical technical drills represented 16.81% of loads in week 12, but only 4.79% in week 14. Also of note, tactically driven drills represented 8.40% of loads in week 12, and 24.55% in week 14. In these weeks, ITM and Foster's Index behaved differently.

More interestingly, though, the average similarities of Foster's Training Monotony and ITM (Figure 3) are quickly revealed to be masking very distinct indices for individual values, which are provided for all players in Figures 4, 5, respectively.

**Figure 6:**
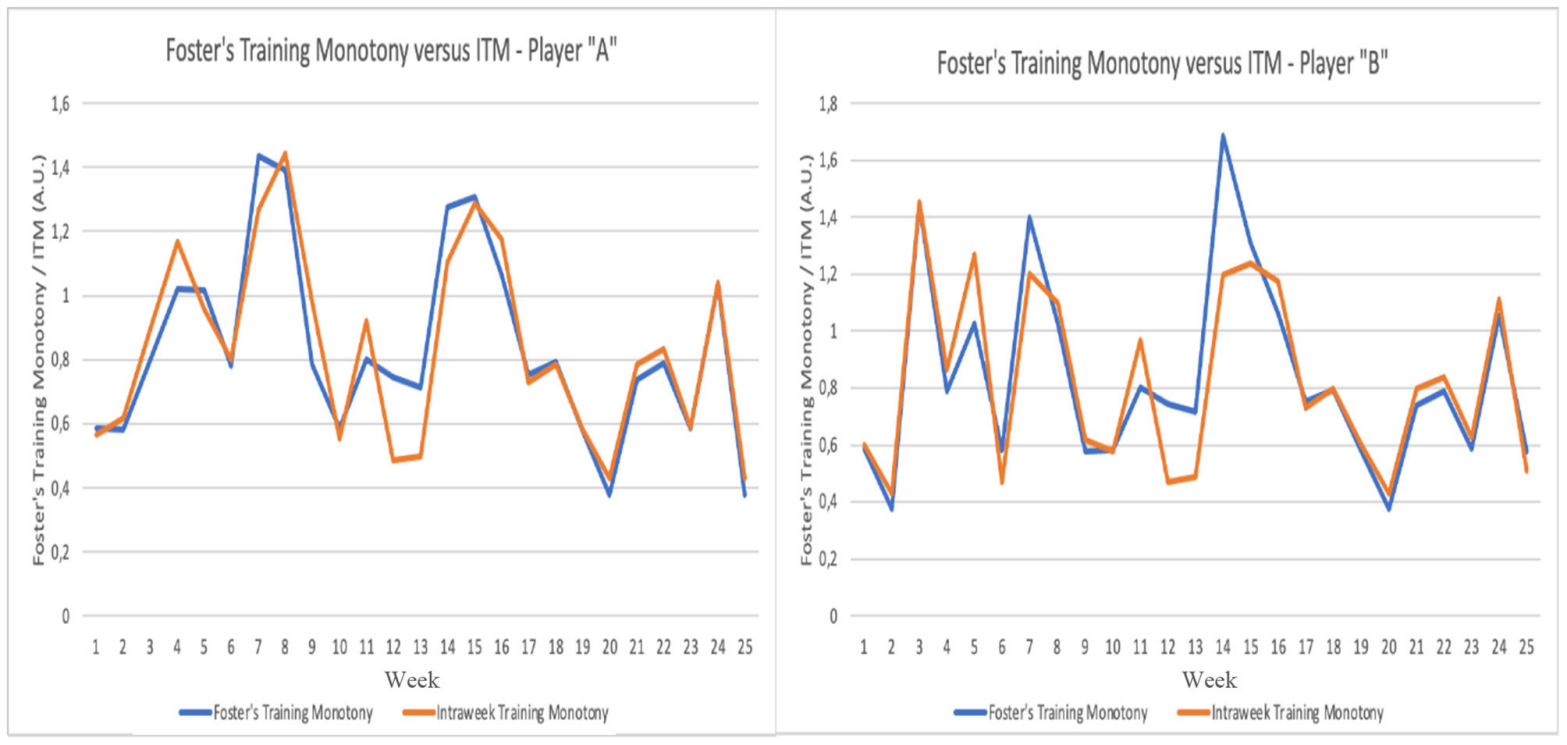
Foster's Training Monotony vs. Intraweek Training Monotony (ITM) for two selected players.

In Figure 6, the data for ITM and Foster's Index is presented for two selected players. The left-side graph depicts the flow of ITM and Foster's Index for Player “A.” The right-side graph depicts the flow of ITM and Foster's Index for Player “B.” The two players were chosen purposefully according to the following criteria: (i) having data points available for all 25 weeks; and (ii) providing qualitatively distinct dynamics of ITM in relation to Foster's Index. While for Player “A,” ITM seemed virtually identical to Foster's Index in terms its qualitative behavior, for Player “B” the behavior of ITM deviated more prominently. For both players, in weeks 12 to 14 ITM deviated considerably from Foster's Index, a trend that had already been analyzed globally. For each player, the residual sum of squares (RSS) was calculated: the smaller this sum, the greater the similarity between the two models. For player “A,” RSS was 0.286. Conversely, for player “B” RSS was 0.555, i.e., for player “B” there was a greater difference between Foster's Monotony Index and ITM. It is interesting to also note week 5: while player “A” exhibited a decreased in ITM, player “B” sharply increased ITM. The raw data showed that players “A” and “B” were exposed to similar load orientations, but player “A” had an exposition to analytical technical drills, a type of load orientation that was absent for player “B” in that particular. This introduced greater heterogeneity for player “A,” contributing for a reduction in monotony. Moreover, in that same week, player “A” had intersession variations in training distance of up to 43%, while in player “B” those intersession variations were limited to a maximum of 22%. These two factors concurred for player “B” to experience a sharp increase in ITM.

**Figure 7:**
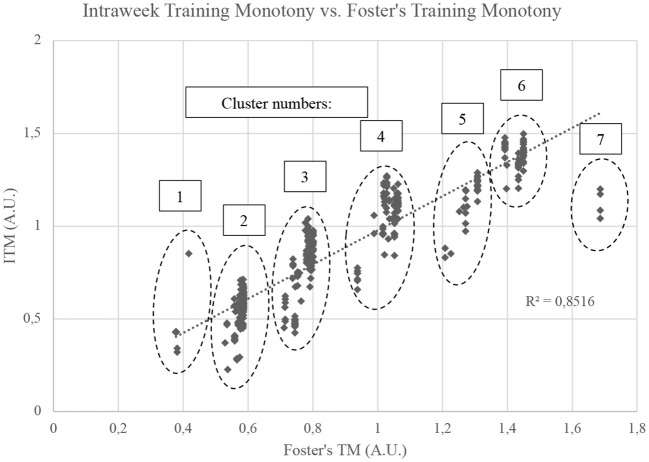
Relationships between Intraweek Training Monotony and Foster's Training Monotony. A.U., Arbitrary units.

The aforementioned differences can also be visualized in Figure 7 and their numerical values presented in Table 2. In Foster's Training Monotony, the data points organize more strongly into clusters, which is expected due its strong reliance of session duration, that will tend to be similar for different players. Although our data does not have sRPE, it was previously established that in Foster's model session duration outweighs sRPE. Furthermore, since sRPE has discrete values, multiplying session duration by sRPE would only have subdivided the clusters and grouping the players with equal sRPE. On the other hand, ITM presents a greater individualization (and scattering) of the obtained values. As dissected in Table 2, the coefficients of variation (CV) of ITM for each cluster of points are superior to the CV of Foster's Training Monotony. So, in each cluster, ITM demonstrates greater variation from player to player than Foster's Index, suggesting that it provides a more highly individualized set of values. In fact, the ratio of ITM CV to Foster's CV varied from 3.000 to 9.769, i.e., ITM's CV was 3 to ~9.8 times superior to that of Foster's Index for each cluster. In cluster 7, all Foster's values were equal, and consequently CV was zero. As such, the ratio of CVs could not be calculated for cluster 7. Therefore, while the average-based analysis suggests that Foster's Training Monotony and ITM convey similar information, individualized analysis show a very different picture. And in this first, simplified application of our model, only total distance was used as a proxy for load, but the model allows introducing combinations of metrics, thereby extending individualization even further.

**Table 2:** Coefficients of variation for Foster's training monotony and intraweek training monotony.

	**Clusters identified in** **Figure 6**						
**Cluster no**.	**1**	**2**	**3**	**4**	**5**	**6**	**7**
Foster's training monotony – CV	0.013	0.017	0.032	0.032	0.024	0.017	0.000
ITM – CV	0.127	0.151	0.208	0.127	0.111	0.051	0.066
ITM/Foster CV Ratio	9.769	8.882	6.500	3.969	4.625	3.000	—^*^

*CV, coefficient of variation; ITM, Intraweek Training Monotony*.

* *In cluster 7, all Foster's values were coincident, providing a CV o zero; therefore, the CV ratio could not be calculated*.

### Acute:Chronic Load

**Figure 8:**
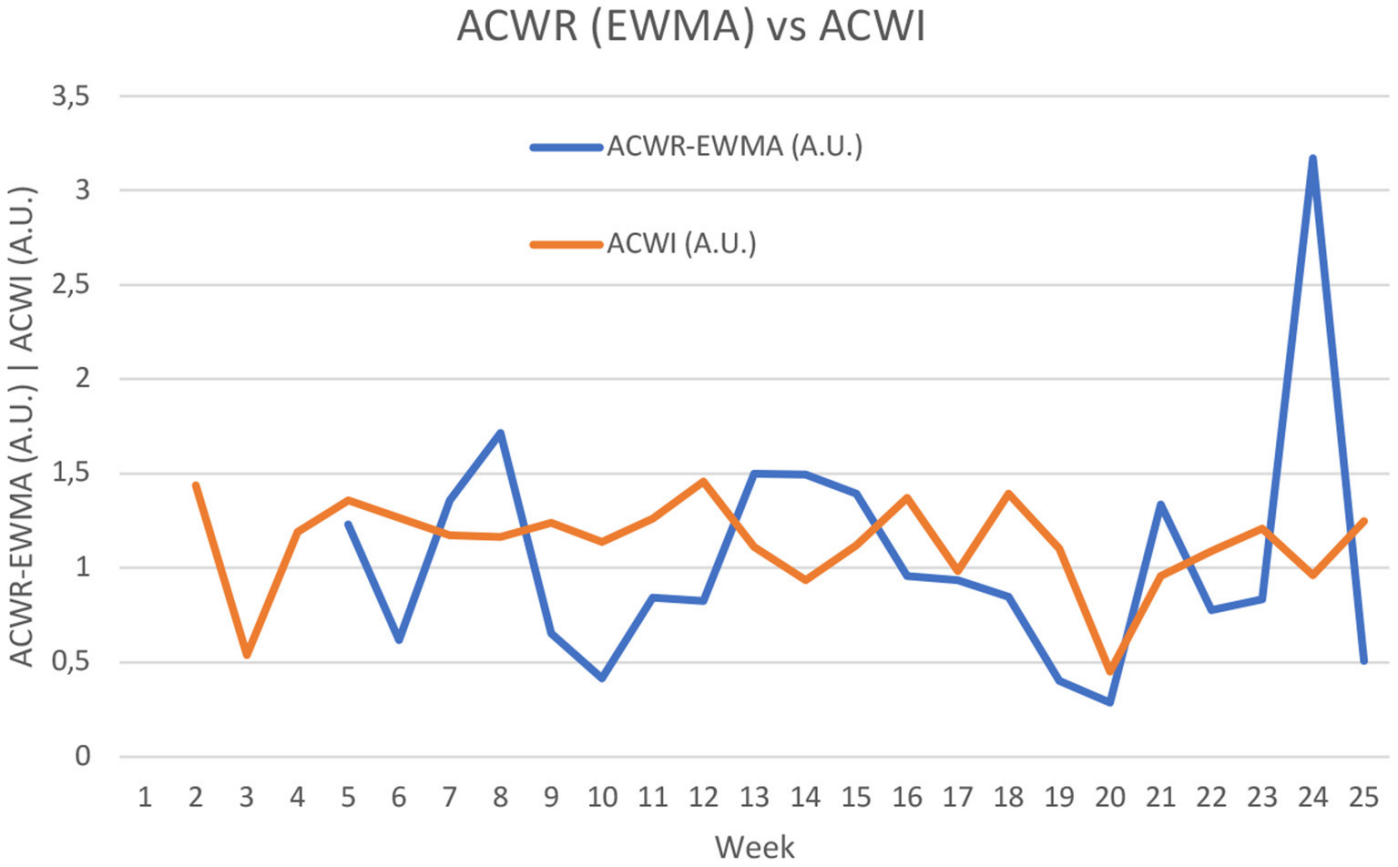
Comparison between Acute:Chronic Workload Ratio (exponentially weighted moving average) and Acute:Chronic Workload Index. A.U., Arbitrary units.

As previously established, the novel ACWI was contrasted to the former uncoupled ACWR with EWMA. In Figure 8, data comparing the ACWR and ACWI is presented. ACWR requires a minimum of 4 chronic weeks, and therefore data can only be calculated starting on week 5. Contrariwise, ACWI accepts any number of chronic weeks, and the week n always presents the greatest weight, followed by n-1, n-2, and so forth. The smaller the weight of any given week in the model, the less the index fluctuates in light of that week's value. For example, if four chronic weeks are considered, the weight of the chronic week, when 4 weeks are considered, is ~40% of total. Starting in the 8th week, the relative weight of the first week in ACWI is reduced to ~22%. The ACWI can therefore be used continuously throughout the season, with no upper limit of weeks that can be used to calculate it.

**Figure 9:**
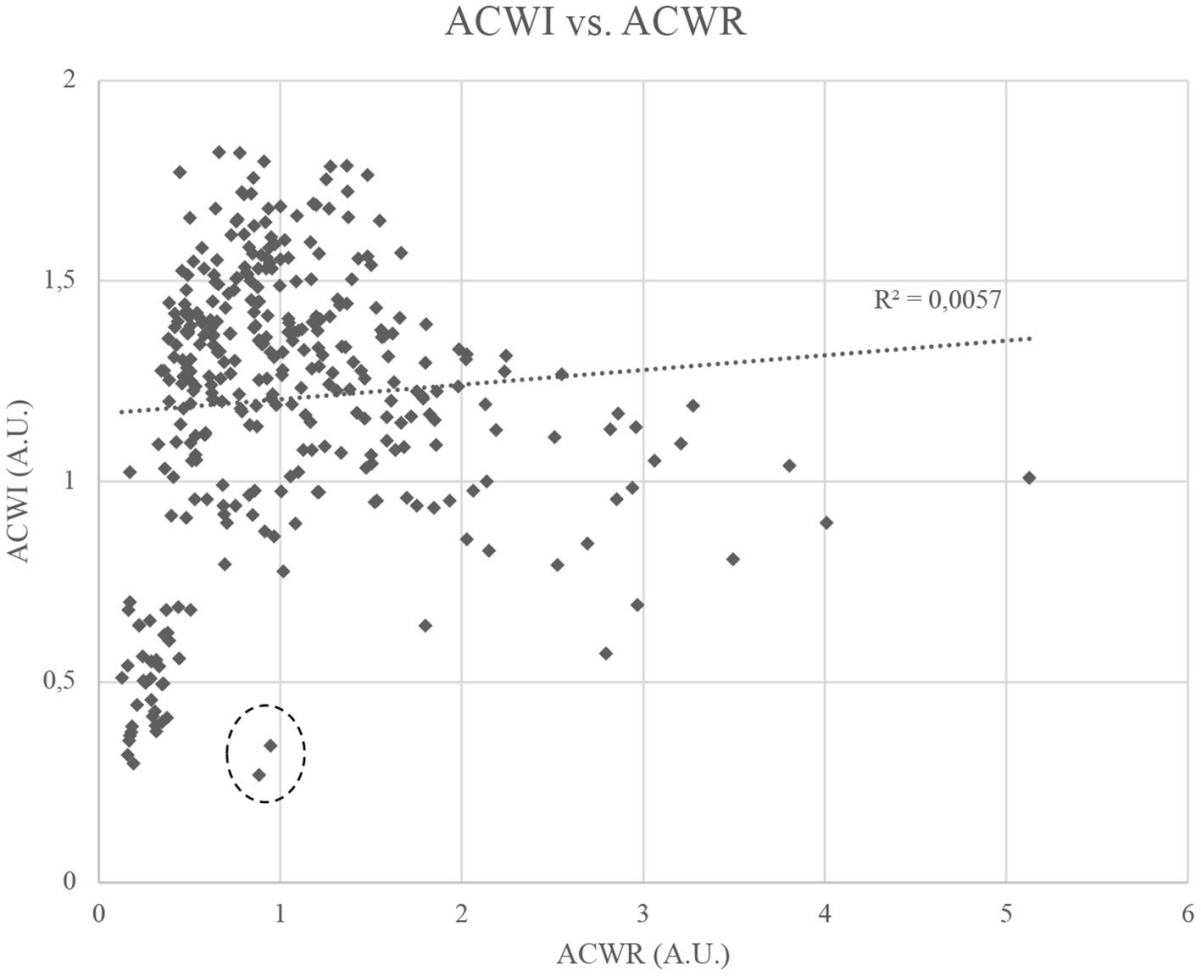
Relationship between Acute:Chronic Workload Ratio (exponentially weighted moving average) (ACWR-EWMA) and Acute:Chronic Workload Index (ACWI). A.U., Arbitrary units.

In both ACWR and ACWI, care should be taken to avoid misinterpreting week to week differences, as in the two models there is influence from previous weeks. For example: in the abrupt changes seen from weeks 23 to 24, ACWI is being influenced by the 22 and 23 previous weeks, respectively. So, analysis of pairs of weeks is not advised. Also, when comparing ACWR and ACWI, there was no meaningful correlation between the models (*r*^2^ = 0.0057) (Figure 9). Although the ACWI values are concentrated between 0 and 2 A.U., they could theoretically reach 3 A.U. The lack of correlation demonstrates that the models are conveying qualitatively different information.

In Figure 9, it is interesting to analyse the two points that were highlighted through a dashed circumference, since they demonstrate how different ACWR and ACWI can be. These two points have an ACWR close to 1, but their ACWI is <0.4. Both these points occurred in week 13, corresponding to two players. One of the players only performed one training session in this week and limited to two training orientations, while in the previous weeks he had an average of three weekly training sessions and performed five training orientations. By assessing load orientation and also weekly density, ACWI was able to capture this very abrupt changes better than ACWR, despite total distance having been similar across these weeks (the player performed only one session, but with very high total distance). For the second player, a similar phenomenon occurred, although with total distance being considerably lower in week 13.

## Discussion

Training is complex, multifactorial endeavor where a fine balance has to be established between load and recovery (Kellmann et al., 2018). Insufficient load will result in poor performance or even detraining, while excessive load will result in poor performance or overtraining (Bompa and Buzzichelli, 2018). Therefore, it is paramount to develop tools that help coaches monitor load dynamics in time, to better adjust their planning and training sessions (Griffin et al., 2020; McGuigan et al., 2020). The problem is that load is a complex construct with many interacting parameters, such as volume, intensity, density, frequency, monotony and complexity (ACSM, 2018; Bompa and Buzzichelli, 2018), but Sports Sciences has focused overly on monitoring volume and intensity (Bradbury et al., 2020). In the last decades, attempts have been made to address more complex load parameters such as Training Monotony (Foster, 1998) and ACWR (Gabbett et al., 2016; Williams et al., 2017). However, these models still rely on raw data limited to proxies of volume and intensity. A particularly relevant load parameter is its orientation: due to training specificity, two loads of the same volume and intensity may generate completely different adaptations depending on their nature (Bompa and Buzzichelli, 2018). Trying to integrate such type of information in workload measures, our goal was therefore to create novel approaches to assessing training monotony and acute:chronic load, considering session duration, training metrics (as proxies of workload), weekly density (frequency normalized) but also bringing load orientation to center stage. In this sense, we have developed new conceptual models that originated specific mathematical approaches, one aiming to assess intraweek training monotony (ITM), the other focusing on a new acute:chronic workload index (ACWI). Because the concepts of hormesis (i.e., the dose-response effect that load variations can have on athletes) and phenotypic plasticity (i.e., the adaptability of an athlete to environmental contexts) will imply inter- and intraindividual variability in response to training stimuli (Kiefer et al., 2018), we propose that our models should be best applied on an individualized basis, with less relevance being attributed to average values. We believe that the new proposed workload measures should be used to control specific training principles as individualization, progressive overload, or variability of stimulus, more than using alternative approaches not related to the dynamics of training stimulus and load (e.g., relationships with injury risk, overreaching or illness).

First and foremost, it should be noted that ACWI is not merely an extension of ITM to more than 1 week. For example, if a coach uses an intraweek training structure that is highly diversified, ITM will be low. But, if the coach repeats that intraweek structure week after week, ACWI will be high. In this particular scenario, AWCR would be close to 1, as acute:chronic workloads would be considered to be stable, but in our ACWI model the values would be high. Conversely, 10 weeks with very high ITMs may compound a low ACWI, as long as those 10 weeks are sufficiently different from each other. Evidently, depending on the planning, the coach may wish to strategically design monotonous training weeks, or even monotonous training periods in some phases of the season (Bompa and Buzzichelli, 2018). Therefore, monotony is not an inherently negative concept. Moreover, it should be highlight that our ACWI can be calculated with as little as one acute week and one chronic week and has no upper limit to the number of chronic weeks than can be included, although the further back in time, the smaller the weight of that specific week.

Our results have shown that the proposed models (ITM and ACWI) provide information that is distinct from previous models for assessing Training Monotony and ACWR, both quantitatively and, most notably, qualitatively. In the case of ITM, the average data is similar to that provided by Foster's Training Monotony, but the individualized analysis provided a very different picture. Possibly, with future expansions of the model and incorporation of more and more diversified proxies of internal and/or external load, ITM will become even more distinct from Foster's ITM. As for ACWR and ACWI, even the average data shows that we are facing two qualitatively very distinct models, and the incorporation of load orientation is a particularity that clearly distinguishes the two models. This, in itself, is relevant, because it confirms that the models are not redundant, instead they provide distinct metrics. For example, ITM seems to provide information that is more sensitive to each player's profile, since it is not overly reliant on session duration and it is able to deal with a more complex set of metrics that better reflect internal and/or external load. From the perspective of the coach, we are bringing to the table a different instrument for monitoring load, where load orientation plays a prominent role and there is greater flexibility in terms of which metrics of load (i.e., proxies of internal and/or external load) can be used. Together, ITM and ACWI can be used to provide a novel understanding of load dynamics and assist coaches in monitoring their training process; while ITM should be applied to a training week, the ACWI represents a long-term model. Finally, it is important to highlight that ACWI can be used continuously throughout the season, i.e., there is no upper limit to the number of weeks that can be input, even if their weight diminishes as time passes. One limitation of our dataset is that no data was available for official matches, and so the calculations were performed using only data from training sessions. However, it is important emphasize that the model allows use both (training sessions and matches).

Our models are not without limitations. In future iterations of the model, sRPE should be integrated in the metrics (which we did not do, since our data were not originally collected with the purpose of testing these models). Furthermore, it might be questioned if session duration is the best metric for assessing session training volume, or if alternative metrics such as intra-training density (Bompa and Buzzichelli, 2018), player training load (Bredt et al., 2020) or concepts exported from pedagogy such as learning time (Siedentop et al., 1982; Whipp et al., 2015) could better reflect the actual daily training volume. Again, future iterations of the model could explore these alternatives. Additionally, the sequencing or ordering of different load directions within the same training session may also be a relevant factor (Sanchez-Sanchez et al., 2018, 2019), but we have not included this factor to avoid excessive complexity. While the factor for metrics of internal and/or external is very open and accepts different inputs, it will most likely be associated with assessments such as percentage of repetition maximum, distance covered in sprint, and other physically dominated parameters. However, this parameter of our model may as well incorporate the cognitive, decisional and emotional impact of load imposed on the athletes (Collins et al., 2018; Ávila-Gandía et al., 2020) and other related concepts. This makes the model customizable and adaptable to the coaches' training philosophy and can allow individual solutions to the athlete preparation puzzle. This is undoubtedly the advantage of the current model in comparison with the previous models, based on pre-defined proxies of load. Additionally, the parameters of the model may evolve in line with the evolution of scientific knowledge, practical experience and technological innovations.

Also, as was previously recognized, our data was not collected with the specific purpose of testing ITM and ACWI, which limits the full testing of these models. However, due to the Covid-19 pandemic, we were unable to collect further data. Still, we felt it would be relevant to expose these new theoretical models to a wider audience, as we are strongly convicted that these ideas may prove useful for Sports Sciences, even if future iterations do not use these specific models. Most importantly, we believe that these models should be used on an individualized basis, avoiding the stipulation of average, arbitrary cut-off values. We know that future research will likely link ITM and ACWI to overall performance and/or injury risk, but again we would advise against the simplistic attempts to find the “magic number.” Beyond inter- and intraindividual variability in response to training, planning will also likely interfere with ITM and ACWI, as different phases of the season tend to have different demands, and these can be by design (Bompa and Buzzichelli, 2018).

We do believe that the main scientific contribution of the new proposed workload measures is related to the capacity of clearly defined variability based on the dimensions of load and structural concept of the drills which was not considered in any other workload measure, as far we know. Additionally, the newly proposed ACWI also provides a good reference for identifying the progression of load, with a strong capacity of integrating any kind of information and without the limitation of getting the previous history of load which is very relevant for particular cases as pre-season or return-to-play after a period of training absence.

## Conclusion

In sum, the ITM and ACWI can be powerful tools in advancing how Training Monotony and Acute:Chronic load are calculated, especially because load orientation plays a relevant role, and also due to the flexibility of which proxies of internal and/or external can be used by the coaches. This provides an approach whereby different coaches and/or different sports can use different proxies of load in their models. However, session duration, weekly density and load orientation will always be considered. Our models try to find a balance between complexity and applicability to a wide range of sports and training conditions. This attempt is likely to be flawed and unfinished but will hopefully provide a more complete account of training monotony than current, narrower approaches. Importantly, this model should be used as an individualized monitoring tool, avoiding the pitfalls of arbitrary cut-off values, whether related to performance or to injury risk. To avoid unsubstantiated applications of both ITM and ACWI, we want to explicitly state that our models are not designed to be used as tools for assessing injury risk. We further invite all readers to actively participate in the improvement of ITM and ACWI, treating them as work in progress, and not as finalized versions.

## Data Availability Statement

The original contributions presented in the study are included in the article/Supplementary Material, further inquiries can be directed to the corresponding author.

## Ethics Statement

The studies involving human participants were reviewed and approved by IPVC-EDL. The patients/participants provided their written informed consent to participate in this study.

## Author Contributions

JA and FC lead the project and wrote and revised the original manuscript. AT, JA, and RS analyzed and interpreted the data and wrote and revised the original manuscript. FN, RC-L, RP, CF, TM, and MF wrote and revised the original manuscript. All authors contributed to the article and approved the submitted version.

## Conflict of Interest

The authors declare that the research was conducted in the absence of any commercial or financial relationships that could be construed as a potential conflict of interest.
