# Supplementary material for: A Novel Approach to Training Monotony and Acute-Chronic Workload Index: A Comparative Study in Soccer
Source: Front Sports Act Living. 2021 May 31;3:661200. doi: 10.3389/fspor.2021.661200 (PMC8200417; doi:10.3389/fspor.2021.661200)
Supplement: Supplementary file 1 [file Data_Sheet_1.zip › Arquivo/2_Mathematical Models.docx]

***Mathematical models***

*Intraweek Training Monotony (ITM) Index*

For the calculation of the ITM, we evaluate the monotony and/or dispersion of each of the four parameters mentioned in the conceptual model: session duration, weekly density, proxies or surrogates of external and/or internal load and inter-session repeatability.

Monotonies of both session duration and session load are evaluated by means of a data dispersion analysis, through the calculation of their coefficient of variation, $C_{V}$. The formulas used for determining the $C_{V}s$ are listed below, with $n$ being the number of sessions in the week.

$$c_{V}=\frac{\sigma}{\bar{x}}, \sigma=\sqrt{\frac{1}{n-1}\sum_{i=1}^{n} \left( x_{i}-\bar{x} \right)^{2}}$$

It would be immediate to take the reciprocal of the coefficient of variation in order to express data monotony, similarly to what is used by Foster in his monotony index; however, its output would result in an unbounded set, ranging from zero to infinity. For this reason, a conventional normalization using Min-Max scaling is impossible to compute and, therefore, not viable. Moreover, for small values of $C_{V}$ (near zero), the inverse function takes increasingly high values. This is undesirable in a model such as ours, consisting of a summation of terms, since the abnormally high value in one term would wrongfully “absorb” the remaining terms, outputting a result with little to no meaning regarding all other monotony parameters. It is also worth noting that when dealing with real values of these metrics, a $C_{V}$ of 1 is rarely exceeded. Given these considerations, we propose a different transformation for the $C_{V}$, other than its reciprocal: by shifting the graph of the reciprocal function, we can obtain a model which outputs 1 when $C_{V}=0$ and outputs 0 when $C_{V}=1$, while maintaining a behavior similar to that of the basic reciprocal function. Although highly unlikely, the value of the coefficient of variation could exceed 1, resulting in a negative value. In this case, the value is truncated to zero. The monotony indicator here described, $M$, is formally defined as:

$$M=\max_{} \left\{ \frac{1-c_{V}}{1+c_{V}}, 0 \right\}$$

Inter-session repeatability establishes a relationship between the number of similar load orientations and the number of distinct orientations between any two training sessions. Therefore, the inter-session repeatability between any two sessions is defined as the fraction of similar load orientations with respect to the total number of orientations approached. An average of the inter-session ratios is calculated dividing their total sum by the number of pairs which can be formed from a total on $n$ sessions, $\binom{n}{2}$. Defining $D_{i}$ and $D_{j}$ as the sets of load orientations engaged in two generic sessions, $i$ and $j$, respectively, the inter-session repeatability between these two sessions, ${Ir}_{ij}$, is defined as:

$${Ir}_{ij}=\frac{\#(D_{i}\cap D_{j})}{\#(D_{i}\cup D_{j})}$$

Lastly, the definition of weekly density used for the purposes of this work consists simply in the division of the number of weekly training session by seven (i.e., the number of days in a week). This parameter alone evaluates the consistency of training throughout the week: the further apart from one, the most days without training there are. The monotony indicators relative to all other parameters will appear multiplied by weekly density, since a high inter-session monotony can be due to a low weekly density. This way, weekly density will reflect the significance of the values of the remaining indicators.

Therefore, and taking into account all previous considerations, the ITM (for a certain week with a total of $n$ training sessions) can be defined as:

$$ITM=\frac{n}{7}\left( M_{duration} +M_{load}+{\binom{n}{2}}^{-1}\sum_{i=1}^{n-1} \sum_{j=i+1}^{n} {Ir}_{ij} \right)$$

*Acute : Chronic Workload Index*

The logic behind the calculation of the ACWI is very similar to that of the ITM. The four domains considered for this inter-week approach are variations in inter-week session durations, variations in weekly density, proxies/surrogates of external and/or internal load, and inter-week repeatability; these parameters correspond to their weekly averages instead of individual sessions. In addition, unlike the ITM, weekly density (the number of weekly training sessions divided by 7) will not be a direct indicator of training monotony; rather, we must determine its inter-week variation/monotony. Another difference relies in the fact that all comparisons of training metrics are made with respect to the acute week. The same principle applies to the comparison between load orientations: the orientations addressed in each chronic week are compared with those addressed in the acute week. Finally, it is worth noting that we use an uncoupled model, and so the acute week is not included in the chronic weeks.

We can first calculate a monotony indicator corresponding to each chronic week, that is, a value reflecting the degree of similarity between a certain chronic week and the acute week. This can be achieved with an approach similar to that of the ITM. For a certain chronic week, $i$, the value for this indicator, ${AC}_{i}$, is defined as follows:

$${AC}_{i}=M_{i,density}\times(M_{i,duration} +M_{i,load}+{Ir}_{ia})$$

Since we are comparing a single pair of values, the use of the coefficient of variation in the calculation of each monotony indicator $M_{i}$ can seem inadequate. However, we can still benefit from its mathematical definition, since it yields a standardized indicator able to assess the degree of variation of a given metric between the two chosen weeks. Defining $\bar{x}_{a}$ as the weekly average of a given metric for the acute week and $\bar{x}_{i}$ as the weekly average of that same metric for a certain chronic week, $i$, the formula for the $c_{V}$ applied to a single pair of values would degenerate into the following indicator, $c_{Vi}^{*}$:

$$c_{Vi}^{*}=\sqrt{2}\frac{|\bar{x}_{i}-\bar{x}_{a}|}{\bar{x}_{i}+\bar{x}_{a}}$$

We can now apply the same definitions used in the ITM to calculate the parameters $M_{i}$ and ${Ir}_{ia}$. With $D_{i}$ and $D_{a}$ representing the sets of load orientations approached in a given chronic week, $i$, and in the acute week, respectively, we define:

$$M_{i}=\max_{} \left\{ \frac{1-c_{Vi}^{*}}{1+c_{Vi}^{*}}, 0 \right\}, {Ir}_{ia}=\frac{\#(D_{i}\cap D_{a})}{\#(D_{i}\cup D_{a})}$$

Finally, the several monotony indicators corresponding to each chronic week, ${AC}_{i}$, are combined by means of an exponentially weighted average (EWA) in order to yield the final value of the ACWI. We can define the iteration formula for the EWA as follows:

$$S_{i}=\left\{ \begin{aligned} {AC}_{1}, &i=1 \\ (1-\beta){AC}_{i}+\beta S_{i-1}, &i>1 \end{aligned} \right.$$

The weighting coefficient $\beta$ for the EWA is commonly defined as $\beta=(n-1)/(n+1)$, with $n$ being the number of chronic weeks. Therefore, for a total of $n$ chronic weeks (the $n^{th}$ week being the one closest to the acute week and the 1^st^ week being the one furthest from the acute week), the ACWI is simply the $n^{th}$ iteration of the previous formula:

$$ACWI= S_{n}$$

***Comparisons***

For our dataset, we compared: (i) ITM with the previous Training Monotony index; and (ii) ACWI with the uncoupled version of ACWR (using EWMA). Qualitative differences between the models were then explored, i.e., trying to understand where they differed and why. In the global analyses, many players have missing values due to injury or absence of training due to diverse reasons. We chose not to exclude such data, because it captures the reality of training processes.
